# Chloroform Syncope Treated by Reversing

**Published:** 1884-11

**Authors:** 


### Chloroform Syncope Treated by Reversing.

As a valuable hint, we note that in the *Brit. Med. Jour.*, Dr. Albert I. Garland relates a case wherein he began to operate on a lady, aged forty-one, for the removal of a scirrhus of the mamma. After examination of the heart, which was found normal, they commenced administering chloroform; but the cardiac action becoming very excited, a mixture of chloroform and ether was used. She was some minutes going under the influence, but there was scarcely any struggling, and the pulse was full, though jerky. He had not finished the incisions round the tumor when she suddenly became livid and the pulse ceased. Artificial respiration was begun, the tongue drawn forward, and strong ammonia applied to the nostrils, without avail. He immediately jumped on the bed, and seizing her legs, raised the body, allowing the the head to touch the bed. In a few seconds, the color returned to the lips, and the pulse to the wrist. Artificial respiration was soon resumed; hot water applied to the region of the heart; and she became sufficiently conscious to speak and to swallow some brandy and ammonia, soon, however, relapsing, pulse and respiration ceasing again. He again reversed, with the same result; but in a short time the syncope returned, and after applying the battery without success, he again reversed, and this time with a satisfactory result, as he was enabled, by the use of the battery and ammonia, to establish reaction.

He considers his case worthy of record, as the successful termination was clearly due to reversing the body, it being impossible, apparently, to stimulate the nerve-centres by any other means; and it is a method of treatment which, he thinks, is not used so often as it deserves to be, judging by the reports of such cases, as he only remembers having seen it mentioned in one instance, and it is one so easily and quickly adopted.

